# Lipid balance must be just right to prevent development of severe liver damage

**DOI:** 10.1172/JCI160326

**Published:** 2022-06-01

**Authors:** Timothy F. Osborne, Peter J. Espenshade

**Affiliations:** 1 Departments of Medicine, Pediatrics, and Biological Chemistry, and Institute for Fundamental Biomedical Research, Johns Hopkins University School of Medicine, St. Petersburg, Florida, USA.; 2 Department of Cell Biology, Johns Hopkins University School of Medicine, Baltimore, Maryland, USA.

## Abstract

Nonalcoholic fatty liver disease (NAFLD) is a major health concern that often associates with obesity and diabetes. Fatty liver is usually a benign condition, yet a fraction of individuals progress to severe forms of liver damage, including nonalcoholic steatohepatitis (NASH) and hepatocellular carcinoma (HCC). Elevated sterol regulatory element–binding protein–driven (SREBP-driven) hepatocyte lipid synthesis is associated with NAFLD in humans and mice. In this issue of the *JCI*, Kawamura, Matsushita, et al. evaluated the role of SREBP-dependent lipid synthesis in the development of NAFLD, NASH, and HCC in the phosphatase and tensin homolog–knockout (PTEN-knockout) NASH model. Deletion of the gene encoding SREBP cleavage–activating protein (SCAP) from the liver resulted in decreased hepatic lipids, as expected. However, SCAP deletion accelerated progression to more severe liver damage, including NASH and HCC. This study provides a note of caution for those pursuing de novo fat biosynthesis as a therapeutic intervention in human NASH.

## Triglyceride and cholesterol biosynthesis

Excess triglycerides (TG) in hepatocytes accumulate as the hallmark feature of nonalcoholic fatty liver disease (NAFLD) and are often accompanied by excess hepatic de novo lipid synthesis driven by sterol regulatory element–binding protein (SREBP) transcription factors (1). Two SREBP genes and 3 protein isoforms exist in mammals, with the SREBP-1c isoform promoting hepatic TG biosynthesis (2). The companion SREBP-1a and SREBP-2 proteins preferentially drive TG and cholesterol biosynthesis, respectively. Because most patients with fatty liver never develop severe liver damage, whether hepatic TG accumulation is a key driver of severe liver disease remains an active area of study.

Phosphatase and tensin homolog (PTEN) deletion drives constitutive signaling through the PI3K/AKT/mTOR pathway, which results in elevated liver TG through the mTOR-dependent increase in SREBP-1c–mediated lipogenesis (3–5). PTEN-knockout (PTEN^ΔL^) mice also ultimately develop nonalcoholic steatohepatitis (NASH) and hepatocellular carcinoma (HCC). However, the role of SREBP-driven lipogenesis in this setting had not been evaluated. SREBP cleavage–activating protein (SCAP) is a polytopic endoplasmic reticulum–localized (ER-localized) escort protein that transports the inactive SREBP precursor from the ER to the Golgi apparatus, where proteases release the active, nuclear-targeted transcription factor from its membrane tether (2). Consistent with its essential role for SREBP function, *SCAP* loss specifically in the liver (designated SCAP^ΔL^) lowers hepatic fatty acid and cholesterol synthesis, but chow-fed SCAP^ΔL^ mice show no pathology on gross examination (6). Notably, liver deletion of *SCAP* also prevents fatty liver in diabetic, high-carbohydrate diet, and high-fat diet rodent models of NAFLD, demonstrating that the SREBP pathway is a therapeutic target for the treatment of NAFLD (7).

## The effects of lipid reduction

In this issue of the *JCI*, Kawamura, Matsushita, et al. (8) report on their combination of the liver-specific PTEN^ΔL^ mouse with a liver-specific SCAP knockout (PTEN/SCAP^ΔL^) to examine the effects of lipid reduction on NAFLD development and the progression to NASH and HCC. Compared with PTEN^ΔL^ mice, PTEN/SCAP^ΔL^ mice showed signs of severe liver disease at only 5 weeks of age, including periportal inflammation and necrosis, which were accompanied by elevated serum levels of liver enzymes and secreted proinflammatory cytokines. The hepatic steatosis evident in PTEN^ΔL^ mice was reduced with the combined knockout of SCAP, consistent with the authors’ expectation. Unexpectedly, by five months, PTEN/SCAP^ΔL^ mice exhibited severe liver fibrosis compared with control PTEN^ΔL^ mice. By seven months of age, all PTEN/SCAP^ΔL^ mice developed HCC tumors, and the number of tumors was approximately 15 times that in comparable PTEN^ΔL^ mice. In an elegant set of experiments, the authors demonstrated that accelerated HCC tumor formation was due to the role of SCAP in SREBP activation. Of particular note, reintroduction of nuclear targeted versions of any one of the three SREBP isoforms reversed the observed liver injury and HCC incidence in PTEN/SCAP^ΔL^ mice.

**Figure 1:**
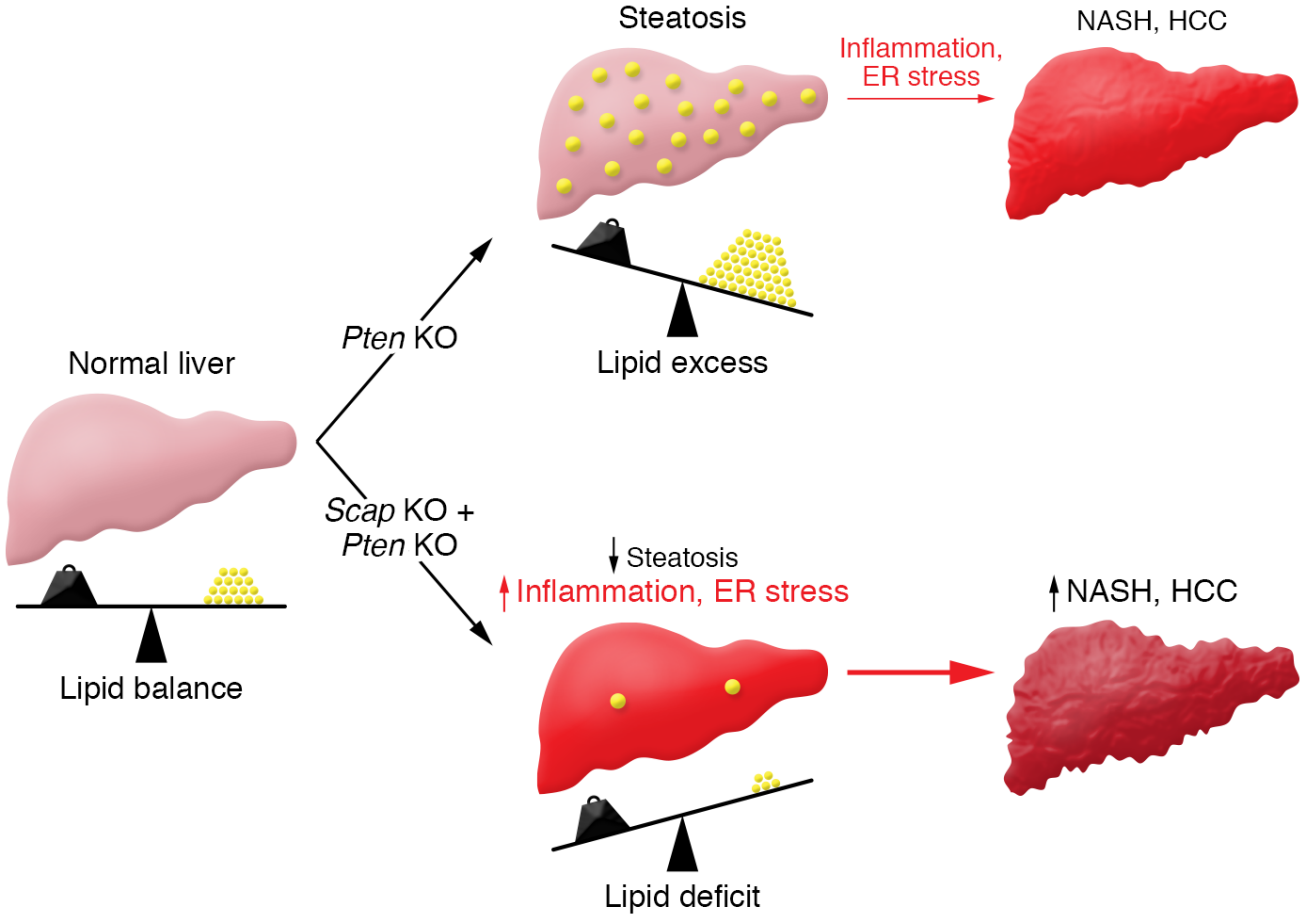
Lipid imbalances in the liver can lead to NASH and HCC progression. Kawamura, Matsushita, et al. (8) showed that a liver-specific PTEN deficiency resulted in excess lipids and steatosis in mice. Notably, PTEN deficiency in the context of SCAP deficiency, which reduces steatosis, exacerbated the later stage development of inflammation and liver injury and accelerated the development of NASH and HCC.

Kawamura, Matsushita, et al. (8) independently tested the effects of SCAP deletion on NASH progression in a second mouse model in which symptoms develop from a choline-deficient, l–amino acid–defined, high-fat diet (CDAHFD) ( 9). In this model, SCAP deletion also resulted in increased liver tumors, indicating that the phenotype was not specific to PTEN deficiency. Interestingly, SCAP deletion also resulted in increased liver damage in response to toxic carbon tetrachloride challenge, showing that SREBP-dependent lipogenesis protected the liver from multiple forms of hepatic toxicity. These results demonstrated that, while liver deletion of SCAP reduced hepatic steatosis, loss of SREBP-dependent lipogenesis sensitized the liver to NASH, which resulted in increased inflammation and HCC carcinogenesis (Figure 1). There was also a positive association in expression of SCAP and SREBPs in patients with NAFLD, providing some correlative evidence that the proposed pathway also has relevancy in human NASH (8).

To investigate the mechanism underlying these unexpected results, Kawamura, Matsushita, and colleagues (8) performed gene-expression and lipidomic analyses on livers from 5-week-old PTEN/SCAP^ΔL^ and PTEN^ΔL^ mice. RNA-Seq comparisons revealed that genes involved in ER stress and the unfolded protein response (UPR) were increased in PTEN/SCAP^ΔL^ mice, and electron microscopy showed morphological defects in ER structure consistent with ER stress. Similar gene-expression signatures were found in isolated hepatocytes from these mice, confirming that the observed ER stress is a cell-autonomous effect due to SCAP deletion. Consistent with ER stress playing a direct role downstream of SCAP deletion, hepatic overexpression of the ER chaperone GRP78 suppressed activation of the UPR marker CHOP and partially rescued liver injury (8). SREBPs also control elongation and desaturation of fatty acids (10). Consistently, lipidomic analyses revealed that long-chain unsaturated fatty acids in phosphatidylcholine (PC) species were dramatically reduced in liver membranes upon SCAP deletion. A more saturated lipid environment is known to decrease membrane fluidity and promote ER stress (11). Supplementation of liposomes enriched for polyunsaturated PC species reversed the ER stress signature in primary hepatocytes from the PTEN/SCAP^ΔL^ mice. Likewise, oral administration of a similar PC cocktail reduced hepatic markers of ER stress in vivo, indicating that SCAP-dependent alterations in ER membrane lipid saturation are a primary cause of hepatocyte ER stress and subsequent liver injury (8).

Because SCAP^ΔL^ mice do not exhibit severe and spontaneous liver damage (6, 7), SREBP pathway–dependent effects on membrane fluidity and ER stress likely cooperate with a specific PTEN-associated process to elicit the observed phenotypes. PTEN deletion elevates signaling through Akt-mTOR, which in turn suppresses autophagy (12). Notably, SREBP-1c and SREBP-2 are both positive regulators of autophagy (13–15). Hepatic expression of the autophagy substrate protein p62 was increased in PTEN/SCAP^ΔL^ mice compared with single-knockout control mice, suggesting that SCAP loss and PTEN loss cooperate to inhibit autophagy flux. In support of reduced autophagy playing a role in PTEN/SCAP^ΔL^ mice, SCAP deletion in mice with a liver-specific knockout of the autophagy gene 5 (ATG5^ΔL^) also increased liver damage and HCC incidence, which was accompanied by elevated ER stress. Thus, inhibition of autophagy via mTOR activation may prevent removal of damaged ER and further exacerbate cell stress created by SCAP deletion. Importantly, liver damage and HCC tumor incidence were less severe in SCAP/ATG5^ΔL^ mice compared with PTEN/SCAP^ΔL^ mice (8), suggesting that other PTEN-dependent functions contribute to the full disease presentation in PTEN/SCAP^ΔL^ mice.

## Broad clinical considerations

SREBPs are central and ubiquitous regulators of lipid homeostasis under normal physiology that are activated to restore lipid supply when these nutrients are limiting (16). In diabetic individuals, elevated insulin drives inappropriate SREBP-dependent lipogenesis, leading to fatty liver, which can progress to NASH and HCC (1). Promisingly, SCAP deletion and the resultant loss of SREBP activity prevent fatty liver in multiple diabetic rodent models (7). Thus, targeting lipid biosynthetic enzymes or the SREBP pathway directly with small molecule inhibitors has been proposed to prevent or treat steatosis before the progression to NASH. Kawamura, Matsushita, et al. (8) demonstrate that complete inhibition of SREBP activity in the PTEN mouse model disrupts cellular membrane lipid homeostasis and induces ER stress. Rather than improve disease, inhibiting SREBPs unexpectedly promoted inflammation and accelerated NASH and HCC progression (Figure 1). Multiple NASH mouse models exist (9), and it will be important to test the effects of SREBP inhibition in a model that replicates the insulin resistance and obesity commonly observed in human NASH. In addition, the authors did not test the effect of heterozygous SCAP deletion, and it is possible that a partial reduction of SREBP activity will improve hepatic steatosis without causing ER stress. Based on Kawamura, Matsushita, et al. (8), strategies that target lipogenesis directly or indirectly through the SCAP/SREBP pathway may be misguided and overly simplistic. Instead, when thinking about targeting lipid metabolism for therapeutic intervention in liver pathology, strategies that target different stages of disease progression will need to be employed to prevent excessive lipogenesis while maintaining membrane lipid homeostasis.

## Version 1 06/01/2022

Electronic publication
